# Abstracts

**Published:** 1915-12

**Authors:** 


### Abstracts.

Several subscribers have said that they considered the Department of Abstracts the most valuable part of the Journal. While we feel that original articles are those that give tone to any magazine, it is obvious that articles subjected to a double process of sifting and with all literary chaff and nonessentials removed, are likely to be of very great value. So far as practical, abstracts requiring it, are illustrated, but it is a great mistake to attach too much importance to pictures. At one period, we were very much impressed with collective abstracts, but the delay in accumulating material and the different view-points and methods of treatment of a subject by different authors, render it very difficult to prepare such abstracts in a way to do justice to each author. Again, recurrence to a subject as it is found in medical literature serves better to refresh the memory and to furnish new thoughts to readers. It is also difficult to plan space and labor so as to cover any one topic at one time. As several times stated, we would appreciate the co-operation of the local profession in making abstracts, not so much to save our own time, for the amount of Abstract matter for which there is space is well within the needs of reading for private professional purposes, but to secure broadness of view, better judgment as to matters outside our own special interest, and because the work of abstracting is good training in itself. We particularly need help in reading Scandinavian, Italian and one very excellent Greek journal. The last can be read by anyone conversant with ancient Greek, and would be well worth the trouble to some young man who does not wish to let his education in classics slip away from him. The task is not so much difficult as slow.

